# Supplementary material for: Bedside functional monitoring of the dynamic brain connectivity in human neonates
Source: Nat Commun. 2021 Feb 17;12:1080. doi: 10.1038/s41467-021-21387-x (PMC7889933; doi:10.1038/s41467-021-21387-x)
Supplement: Supplementary file 1 — Supplementary Information [file 41467_2021_21387_MOESM1_ESM.pdf]

Supplementary Information

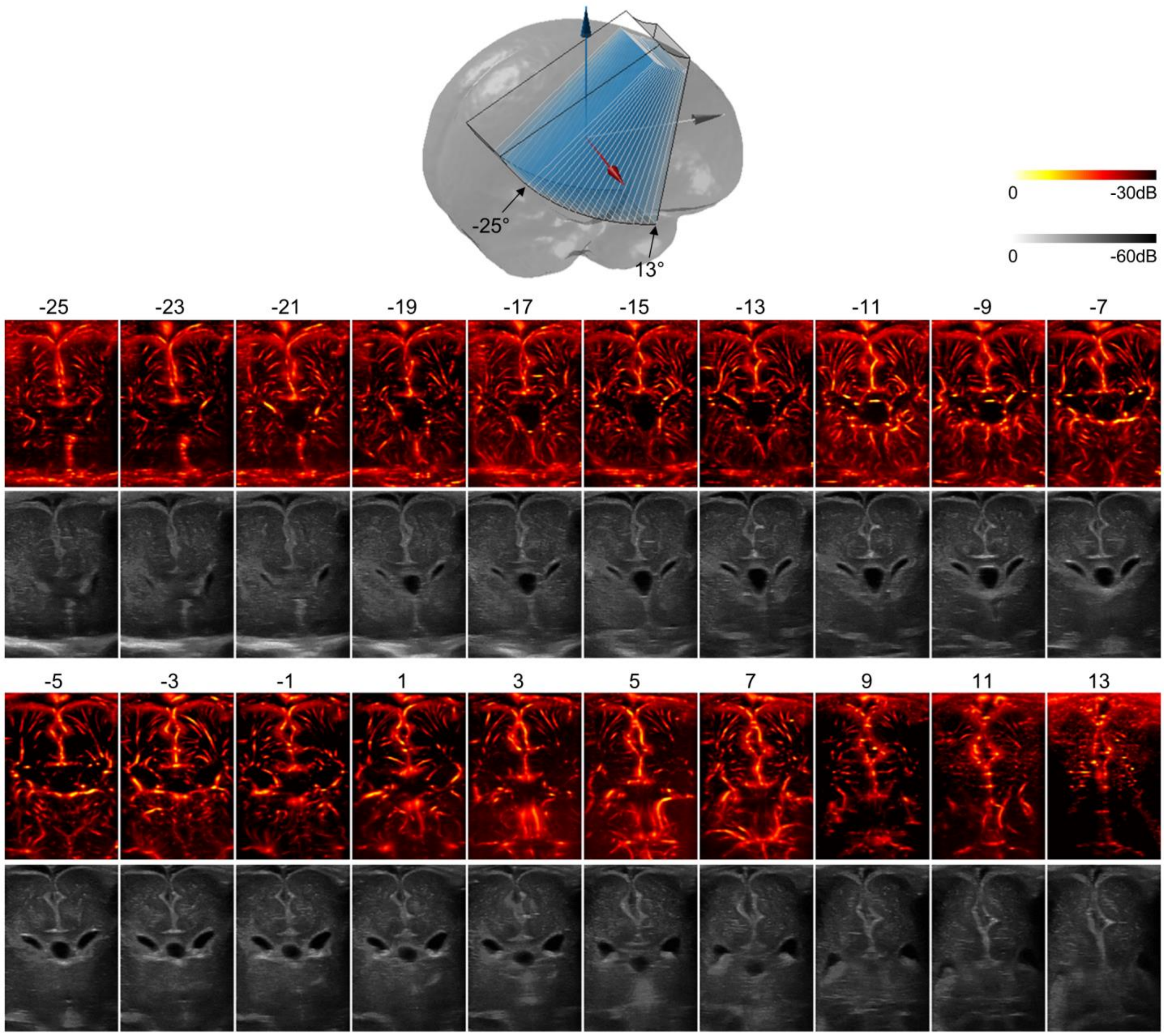

Fig. S1 Plane-by-plane coronal scan using the motorized probe. Top panel: 3D position of the 20 selected planes, linearly steered between -25 to 13 degree. Bottom panel: 20 coronal slices with both Ultrafast Doppler (above) and focused B-Mode (below).

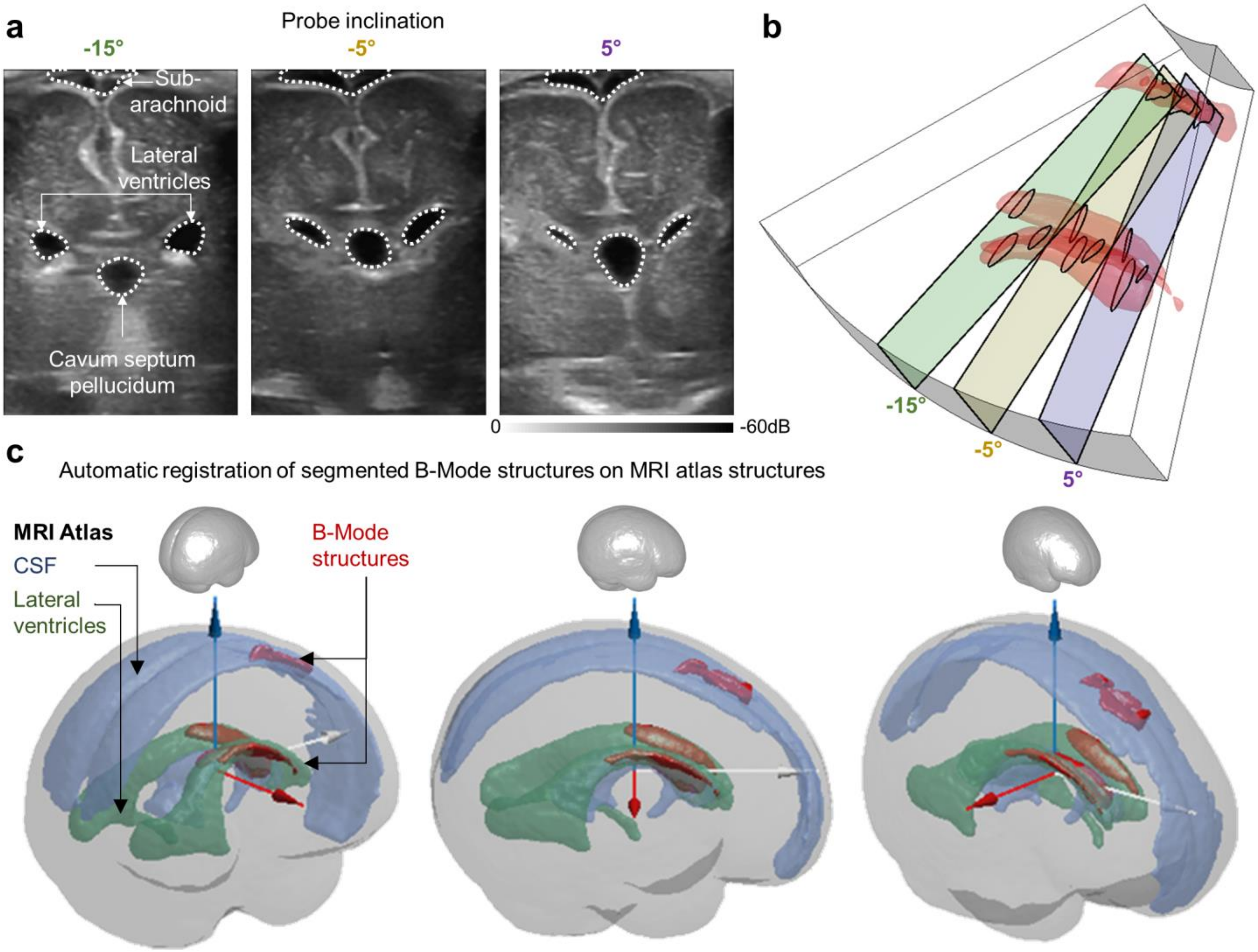

Fig. S2 3D registration of B-Mode volume over MRI atlas using CSF-filled structures. (CSF = cerebrospinal fluid) **a)** Identification of 3 hypoechoic CSF-filled structures in 3 different B-Mode coronal slices. The sub-arachnoid space, the lateral ventricles and the cavum septum pellucidum are automatically segmented. **b)** 3D reconstruction of the B-Mode CSF-filled structures (red) from their sections segmented in coronal slices (green, yellow and purple planes, black contours). **c)** Final registration of the B-Mode-segmented CSF volume (red) over the MRI-segmented CSF volume (blue: MRI sub-arachnoid space and cavum septum pellucidum, green : MRI cerebral ventricles). 3 different views are shown. The MRI T1 whole brain volume is superimposed in light grey for better visualization.

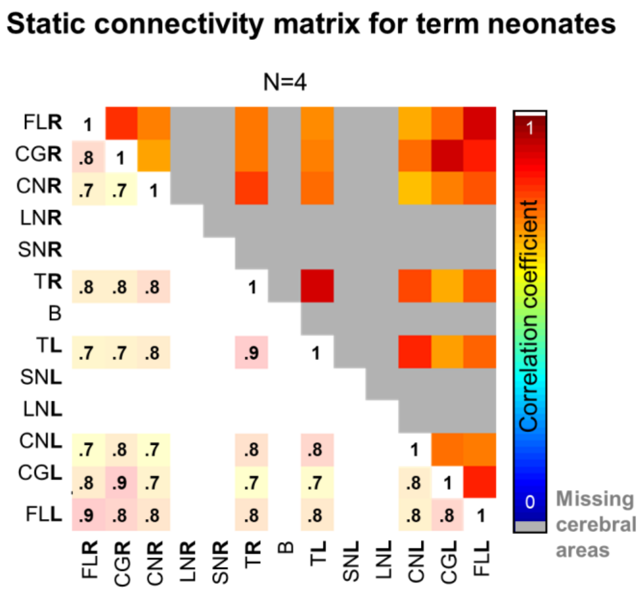

Fig. S3 Static connectivity matrix for term neonates (N=4). For term neonates, due to the brain growth, some areas were not visible in the probe field of view, as compared to preterm neonates. These missing cerebral areas are represented in grey.

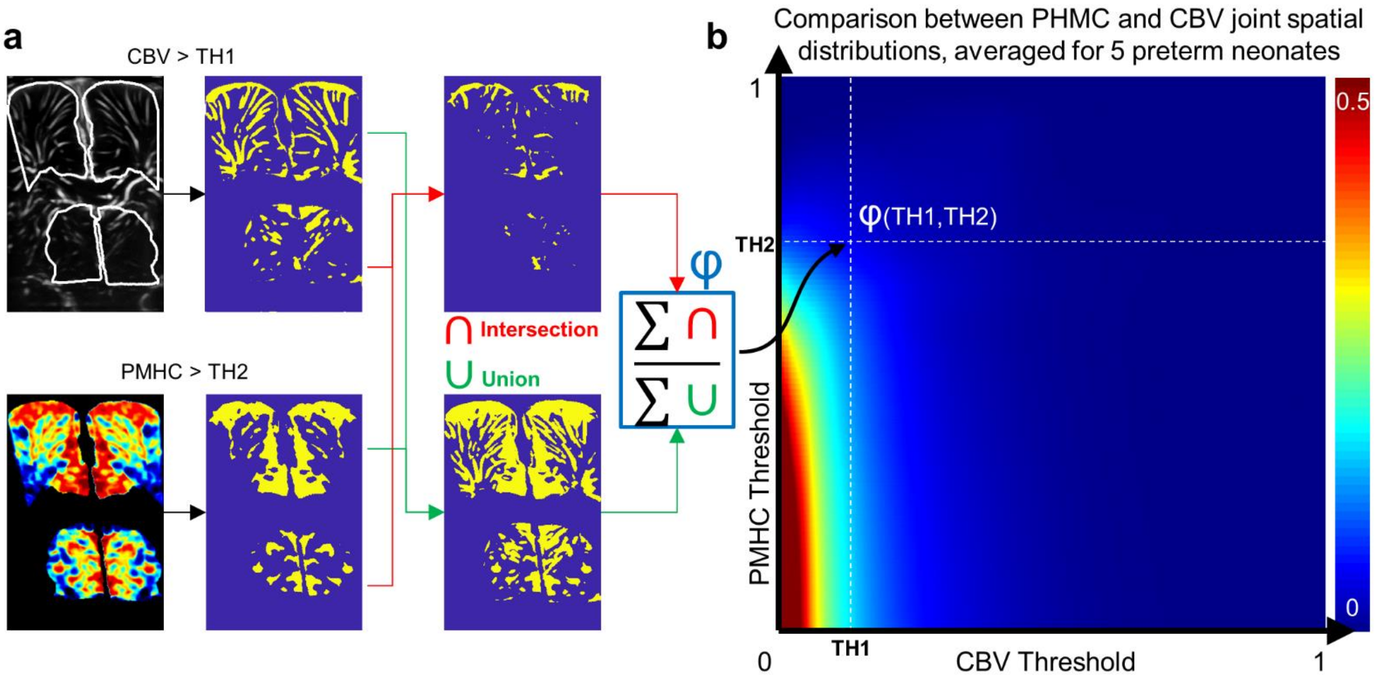

Fig. S4 PMHC high-values are not correlated to high CBV values. (PMHC = Pixel-Mirrored Homotopic Connectivity, CBV = cerebral blood volume) **a)** For each patient, the CBV map is log-compressed in the range [-30 0] dB then normalized. Only the PMHC region of interest is considered (white lines). The resulting CBV map and the PMHC map are thresholded respectively by TH1 and TH2, resulting in two binary masks. The union and the intersection of these two masks are computed. The ratio of the area of the intersection over the area of the union, ϕ, quantifies how much the spatial distribution of CBV and PMHC are linked to each other for this particular pair of thresholds (TH1,TH2). **b)** All the pairs (TH1,TH2) are tested and the results are averaged over 5 preterm neonates. The most interesting part of the graph is when TH2 reaches high values, typically above 0.5. In this case, whatever the value of TH1 is, the ϕ ratio remains low. This translates the fact that there is no obvious link between the spatial distribution of high PMHC values and the underlying CBV magnitude. One can easily verify that these ϕ values are lower than what would be obtained if PMHC and CBV were purely random variables.

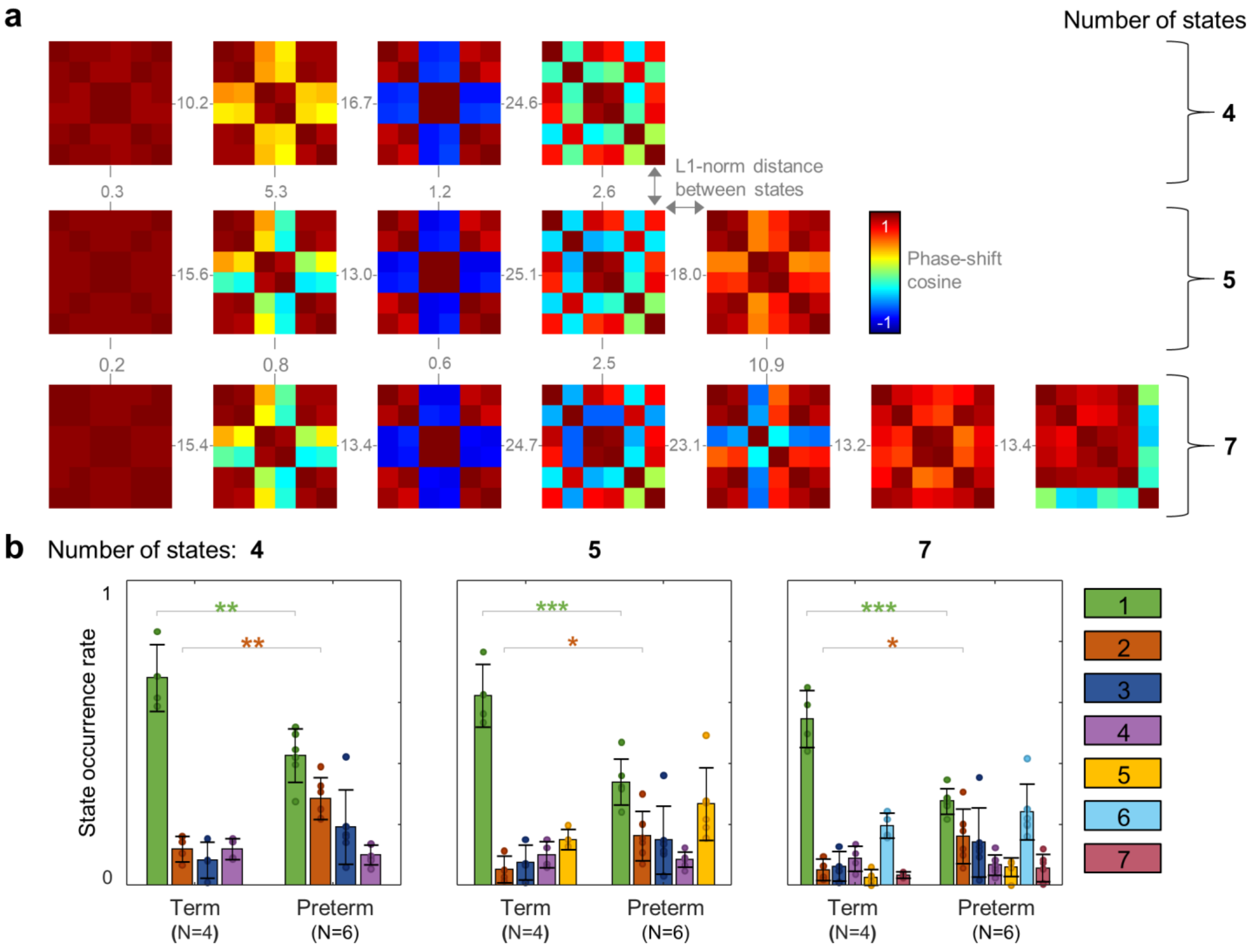

Fig. S5 Influence k-means states number on the dynamic connectivity results interpretation. (PMHC = Pixel-Mirrored Homotopic Connectivity, CBV = cerebral blood volume) **a)** 3 output of the k-means algorithm over phase matrices (see Fig. 4), with respectively k=4, 5 and 7 states requested. The L1-distance between states is added in light gray. For 4x4 matrices, L1 varies between 0 and 16. The 4 first states are stables and remain relatively unchanged (L1 = 0.7±1.1) for every value of k. **b)** State occurrence rate for different value of k. The main trend is conserved whatever the value of k. In particular, state #1 and state #3 occurrence rate remain significantly different between the group term/preterm infants and the burst-suppression case (* $p < 0.05$, ** $p < 0.01$, one-way ANOVA with post-hoc Tukey's tests, from left to right: p=0.003, p=0.003, p=0.001, p=0.039, p=0.0001, p=0.050). N = number of independent patients in the group, with one acquisition per patient.

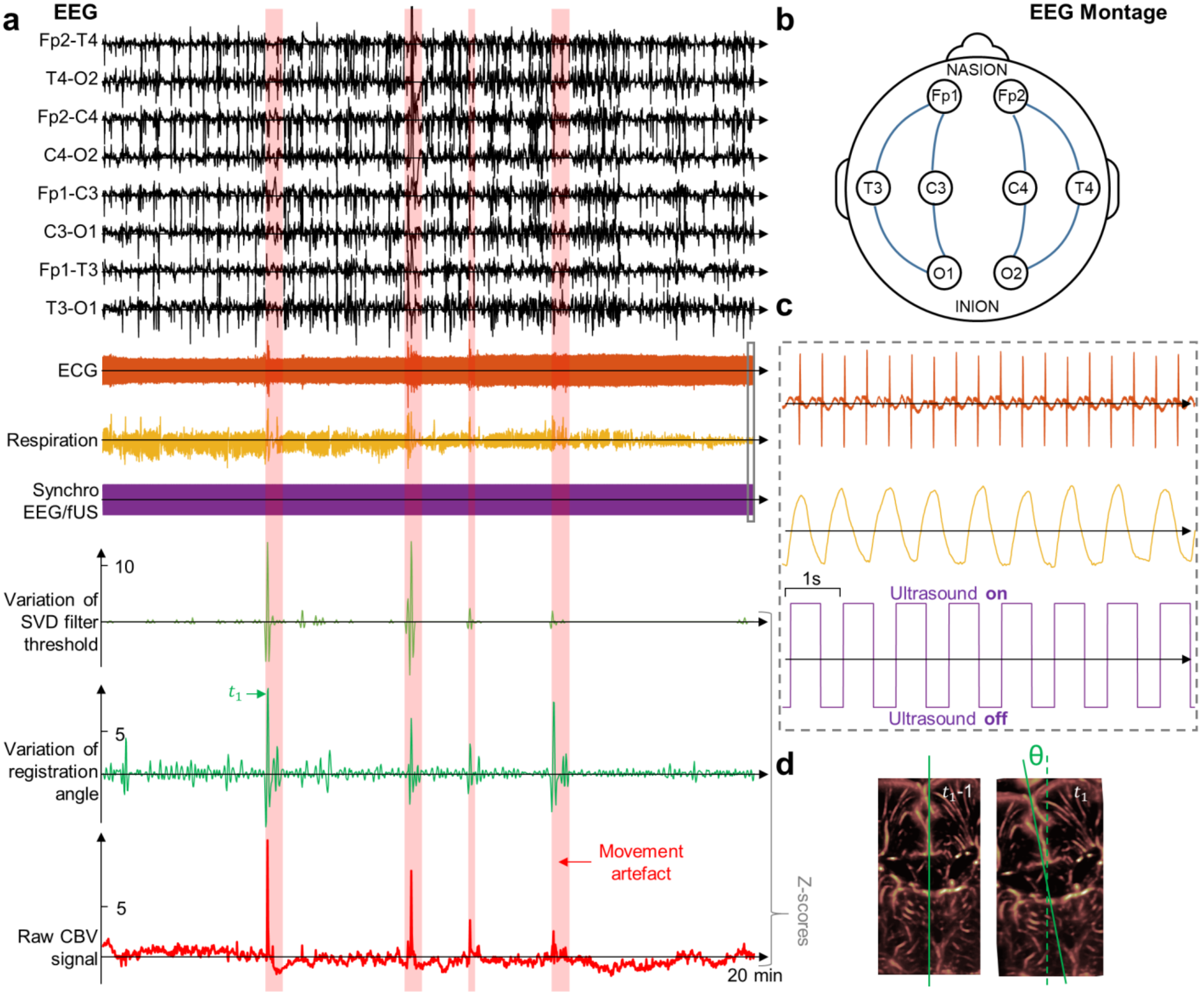

Fig. S6 Signals synchronization and motion artifacts rejection. (SVD = singular value decomposition, CBV = cerebral blood volume) **a)** The 8-electrode EEG **b)**, the ECG and the respiration are recorded on the same device. They are synchronized to the ultrasound signal through a trigger channel **c)**. The motions artifacts are automatically suppressed from the CBV signal by detecting peaks in the adaptive SVD thresholds and by considering the registration angle shift between consecutive frames, illustrated in panel (d).
