# Supplementary material for: Bedside functional monitoring of the dynamic brain connectivity in human neonates
Source: Nat Commun. 2021 Feb 17;12:1080. doi: 10.1038/s41467-021-21387-x (PMC7889933; doi:10.1038/s41467-021-21387-x)
Supplement: Supplementary file 3 — Description of Additional Supplementary Files [file 41467_2021_21387_MOESM3_ESM.docx]

**Description of Additional Supplementary Files**

File Name: **Supplementary Movie 1: Ultrasound plane-by-plane tomography combining a coronal scan and sagittal scan.**

Description: Two scans are performed consecutively in coronal (left panel) and sagittal view (middle panel). The probe steering is motorized but it has to be rotated manually from coronal view to sagittal view. Both scans are registered and merged into a single volume (right panel).

File Name: **Supplementary Movie 2: Automated registration of ultrasound date into an MRI cerebral atlas.**

Description: See movie captions.

File Name: **Supplementary Movie 3: Overview of the brain volume accessible with the current ultrasound sequence.**

Description: The MRI T1 full brain volume appears in grey. In lighter gray the trapezoid volume underlines the boundaries of the volume accessible with ultrasound using the setup described in this manuscript. The 3D Doppler vasculature is overlayed in red.

File Name: **Supplementary Movie 4:** **Construction of the Pixel-Mirrored Homotopic Connectivity map for a given patient.**

Description: Left panel: For each position of a seed pixel in the right hemisphere (blue dot), the mirrored homotopic pixel is identified in the contra-lateral hemisphere (red dot). Right panel: for each pair of homotopic pixels, the Pearson correlation coefficient *r* is computed between the associated CBV time courses. *r* is then displayed in a color scale.

File Name: **Supplementary Movie 5: Dynamic connectivity variations for one preterm neonate and one term neonate.**

Description: Left panels: L1-norm trace (black lines) and timeline of the closest connectivity state (color ribbon). Four connectivity states were considered, each one being encoded by the color used in Fig.4 for similar purpose. Central panel: current phase matrix for each patient. Right panel: dynamic connectivity represented as links between the 6 considered cerebral structures (frontal lobe left/right, cingulate gyrus left/right, thalamus left/right). The color and thickness of the links varies according to the phase-shift cosines between the corresponding areas. Qualitatively, the preterm neonate exhibits more occurrence of talamo-cortical disconnections.
