# Supplementary material for: Bedside functional monitoring of the dynamic brain connectivity in human neonates
Source: Nat Commun. 2021 Feb 17;12:1080. doi: 10.1038/s41467-021-21387-x (PMC7889933; doi:10.1038/s41467-021-21387-x)
Supplement: Supplementary file 9 — Reporting Summary [file 41467_2021_21387_MOESM9_ESM.pdf]

nature research

Corresponding author(s): Jerome Baranger
Olivier Baud
Mickael Tanter

Last updated by author(s): Dec 17, 2020

Reporting Summary

Nature Research wishes to improve the reproducibility of the work that we publish. This form provides structure for consistency and transparency in reporting. For further information on Nature Research policies, see our Editorial Policies and the Editorial Policy Checklist.

Statistics

For all statistical analyses, confirm that the following items are present in the figure legend, table legend, main text, or Methods section.

n/a	Confirmed
☐	☒ The exact sample size (*n*) for each experimental group/condition, given as a discrete number and unit of measurement
☐	☒ A statement on whether measurements were taken from distinct samples or whether the same sample was measured repeatedly
☐	☒ The statistical test(s) used AND whether they are one- or two-sided<br>*Only common tests should be described solely by name; describe more complex techniques in the Methods section.*
☐	☒ A description of all covariates tested
☐	☒ A description of any assumptions or corrections, such as tests of normality and adjustment for multiple comparisons
☐	☒ A full description of the statistical parameters including central tendency (e.g. means) or other basic estimates (e.g. regression coefficient) AND variation (e.g. standard deviation) or associated estimates of uncertainty (e.g. confidence intervals)
☐	☒ For null hypothesis testing, the test statistic (e.g. *F*, *t*, *r*) with confidence intervals, effect sizes, degrees of freedom and *P* value noted<br>*Give P values as exact values whenever suitable.*
☐	☒ For Bayesian analysis, information on the choice of priors and Markov chain Monte Carlo settings
☐	☒ For hierarchical and complex designs, identification of the appropriate level for tests and full reporting of outcomes
☐	☒ Estimates of effect sizes (e.g. Cohen's *d*, Pearson's *r*), indicating how they were calculated

Our web collection on statistics for biologists contains articles on many of the points above.

Software and code

Policy information about availability of computer code

Data collection	The commercial Supersonic Imaging Aixplorer API was used for data acquisition.
Data analysis	All data analysis are performed using the commercial software Matlab 2017b from MathWorks, with the signal processing toolbox, the image processing toolbox and the statistical analysis toolbox.

For manuscripts utilizing custom algorithms or software that are central to the research but not yet described in published literature, software must be made available to editors and reviewers. We strongly encourage code deposition in a community repository (e.g. GitHub). See the Nature Research guidelines for submitting code & software for further information.

Data

Policy information about availability of data

All manuscripts must include a data availability statement. This statement should provide the following information, where applicable:

- Accession codes, unique identifiers, or web links for publicly available datasets
- A list of figures that have associated raw data
- A description of any restrictions on data availability

All data that support the findings of this study are available from the corresponding author (MT) upon reasonable request. For figure 4 and 5, the source data are provided with this paper.

Field-specific reporting

Please select the one below that is the best fit for your research. If you are not sure, read the appropriate sections before making your selection.

☒ Life sciences ☐ Behavioural & social sciences ☐ Ecological, evolutionary & environmental sciences

For a reference copy of the document with all sections, see nature.com/documents/nr-reporting-summary-flat.pdf

Life sciences study design

All studies must disclose on these points even when the disclosure is negative.

Sample size	9 very preterm and 4 term-born neonates were included in the study. The rationale for this cohort size was dictated by the limited access to patients in the complex environment of neonatal intensive care unit. 1 neonate with a congenital "burst-suppression" pathology was included. The very scarce occurence of such clinical cases explains the size of that group.
Data exclusions	3 very preterm neonates were not included in the connectiviy analysis because the mechanical setup (probe holding headset) was reworked after these first trials.
Replication	The scans were performed by a trained neonatalogist that was able to identify the same plane of interest for each neonate. Interhemispheric connectivity was successfully identified for each very preterm and term-born neonate. Only one 20min-long acquisition was acquired for each preterm and term-born patient. For the pathological case, three independent acquisitions were performed on two different days and exhibited the same results.
Randomization	For this proof of concept study, the patients were not randomly assigned to groups and these were constituted based on the gestational age. The last group consisted of a single pathological patient.
Blinding	The sleep phases of the neonates were blindly selected. A trained physician identified the sleep phases on the EEG of all neonates and blindly transferred them to the physicist for the connectivity analysis. However, the connectivity analysis implied a manual-tuning step to finely register the MRI atlas on the ultrasound data. This step was performed by the physicist as it requires technical programming skills at the current stage of development. By doing so, the morphology of the patient was revealed to the physicist, who could hence identify if the patient was preterm or term-born, due to obvious changes of brain size. Consequently, the connectivity analysis cannot claim to be performed in a fully blinded manner.

Reporting for specific materials, systems and methods

We require information from authors about some types of materials, experimental systems and methods used in many studies. Here, indicate whether each material, system or method listed is relevant to your study. If you are not sure if a list item applies to your research, read the appropriate section before selecting a response.

Materials & experimental systems

n/a	Involved in the study	
☒	☐	Antibodies
☒	☐	Eukaryotic cell lines
☒	☐	Palaeontology and archaeology
☒	☐	Animals and other organisms
☐	☒	Human research participants
☒	☐	Clinical data
☒	☐	Dual use research of concern

Methods

n/a	Involved in the study	
☒	☐	ChIP-seq
☒	☐	Flow cytometry
☒	☐	MRI-based neuroimaging

Human research participants

Policy information about studies involving human research participants

Population characteristics	9 very preterm neonates (5 males and 4 females) born at 28±2 weeks PMA were included in the study. The 3 first patients helped to adjust the fUS headset design and therefore were not included in the quantitative study. 4 term-born neonates (2 males and 2 females) in their first week of life were also included. Finally, 1 pathological term newborn was imaged as well. This patient had a KNCQ2 encephalopathy whose symptoms were frequent seizures and a "bust-suppression" EEG pattern.
Recruitment	The patients were recruited by the physician of the neonatal intensive care unit depending of their gestationnal age. The parents guardians were informed of the aim and the principles of the protocol. They then gave a written consent.
Ethics oversight	Protocol approved by the ethical board of Robert Debré Hospital (Comité de Protection des Personnes #120601, BELUGA protocol) and promoted by INSERM (Institut National de la Sante et de la Recherche Medicale), French Health Institute.

Note that full information on the approval of the study protocol must also be provided in the manuscript.
